# Frequency-differencing strategy to kickstart full-waveform inversion without cycle skipping

**DOI:** 10.1121/10.0034763

**Published:** 2025-01-03

**Authors:** Rehman Ali, Trevor Mitcham, Israel Owolabi, Sarah McConnell, Nebojsa Duric

**Affiliations:** 1 Department of Imaging Sciences, University of Rochester, Rochester, New York 14642, USA; 2 Department of Electrical and Computer Engineering, University of Rochester, Rochester, New York 14642, USA; 3 Department of Neuroscience, University of Rochester, Rochester, New York 14642, USA Rehman_Ali@URMC.Rochester.edu, Trevor_Mitcham@URMC.Rochester.edu, iowolabi@UR.Rochester.edu, Sarah_McConnell@URMC.Rochester.edu, Nebojsa_Duric@URMC.Rochester.edu

## Abstract

Ultrasound tomography fundamentally relies on low-frequency data to avoid cycle skipping in full-waveform inversion (FWI). In the absence of sufficiently low-frequency data, we can extrapolate low-frequency content from existing high-frequency signals by using the same approach used in frequency-difference beamforming. This low-frequency content is then used to kickstart FWI and avoid cycle skipping at higher frequencies. In simulations, the structural similarity index measure and peak signal-to-noise ratio of the reconstructed image improve by 0.28 and 8.6 dB, respectively, as a result of frequency differencing. Experiments show that internal structures can be seen with greater clarity because of frequency differencing.

## 1. Introduction

Ultrasound tomography (UST) uses the transmission of sound through tissue to reconstruct high-resolution images of tissue properties such as sound speed and attenuation. The main clinical application of UST is breast imaging (Duric *et al.*, 2007; Li *et al.*, 2009; Wiskin *et al.*, 2012) where sound speed and attenuation serve as biomarkers for cancer detection. Because the full-waveform inversion (FWI) algorithm used in UST directly minimizes the error between simulated and measured signals, FWI often requires low-frequency signals (<1 MHz) to overcome cycle skipping caused by errors in the starting model (Ali *et al.*, 2023). If the simulated signal is shifted by more than a half cycle relative to the measured signal, the underlying model used in the simulation will update in the wrong direction because of cycle skipping. Low frequencies lengthen this half-cycle window, allowing FWI to overcome errors in a starting model. Newer applications of UST such as transcranial imaging (Marty *et al.*, 2024; Mitcham *et al.*, 2024) often require starting FWI at even lower frequencies.

Unfortunately, most ultrasound transducers are driven to higher frequencies by the need to maximize the resolution of B-mode reflectivity imaging. Because FWI requires low frequencies to overcome cycle skipping, it is often difficult to apply FWI to data from the same high-frequency hardware. Therefore, the lack of low-frequency signals from most B-mode ultrasound imaging transducers prevents FWI from becoming more broadly applicable. One way to overcome this limitation is to synthesize the necessary low-frequency content from the available high-frequency ultrasound signals by using the frequency-differencing approach from sonar (Abadi *et al.*, 2012). In traditional FWI, reconstruction can begin either from a homogeneous or a ray-based starting model. The homogeneous model requires low frequencies that may not be available while the ray model can lead to inherited artifacts in the final reconstruction (Ali *et al.*, 2023). Our proposed approach generates a starting model that overcomes these disadvantages. By taking the ratio of signals at two different frequencies *f*_1_ and *f*_2_, we generate a new signal at a frequency $\Delta f=\left|f_{1}-f_{2}\right|$. If Δf is low enough, FWI can be applied to the resulting data to generate a starting model that helps overcome cycle skipping at higher frequencies.

## 2. Theory

### 2.1 Frequency-domain FWI

The objective function for least squares minimization in frequency-domain FWI at a frequency ω=2πf is

$$E(\omega ,s)=\frac{1}{2}\sum_{i=1}^{N}{\left|\left|p_{i}(\omega ,s)-p_{\text{obs},i}(\omega )\right|\right|}_{2}^{2},$$ (1)

where $p_{i}$ and $p_{\text{obs},i}$ are the simulated and observed pressure measurements for the transmission from the *i*th element on the ring array. For complete details on how this minimization is performed (including the conjugate gradient algorithm, source estimation, and data pre-processing), see our prior work (Ali *et al.*, 2024).

### 2.2 Frequency-differencing

Frequency-differencing over the band $\omega \in [\omega_{\text{low}},\omega_{\text{high}}]$ yields the signal ${\bar{p}}_{\text{obs},i}(\Delta \omega )$ at frequency difference $\Delta \omega \ll \omega_{\text{low}},$

$${\bar{p}}_{\text{obs},i}(\Delta \omega )=\frac{\int_{\omega_{\text{low}}}^{\omega_{\text{high}}}p_{\text{obs},i}(\omega +\Delta \omega )⊙p_{\text{obs},i}^{*}(\omega )d\omega }{\int_{\omega_{\text{low}}}^{\omega_{\text{high}}}p_{\text{obs},i}(\omega )⊙p_{\text{obs},i}^{*}(\omega )d\omega }.$$ (2)

The implicit assumption behind Eq. (2) is that the pressure signal $p_{\text{obs},i}(\omega )$ has a linear phase corresponding to some time of flight *τ_i_* [i.e., $p_{\text{obs},i}(\omega )\propto e^{j\omega \tau_{i}}$]. If $e^{j\omega \tau_{i}}$ is substituted for $p_{\text{obs},i}(\omega )$, then ${\bar{p}}_{\text{obs},i}(\Delta \omega )=e^{j\Delta \omega \tau_{i}}$. In other words, Eq. (2) will extrapolate the linear phase to a desired frequency Δω. However, tissue heterogeneity will cause the measured data to deviate from this linear phase assumption because of diffractive effects. In practice, as long as the tissue heterogeneity is reasonably small (e.g., as in soft tissues), the deviation from the linear phase assumption can be managed. At this point, we can use the standard frequency-domain FWI algorithm with the following objective function:

$$E(\Delta \omega ,s)=\frac{1}{2}\sum_{i=1}^{N}{\left|\left|p_{i}(\Delta \omega ,s)-{\bar{p}}_{\text{obs},i}(\Delta \omega )\right|\right|}_{2}^{2}.$$ (3)

This objective function [Eq. (3)] is identical to the original FWI objective function [Eq. (1)], except that Δω replaces *ω* and ${\bar{p}}_{\text{obs},i}(\Delta \omega )$ replaces $p_{\text{obs},i}(\omega )$. In other words, a single Helmholtz equation at frequency Δω is used to simulate $p_{i}(\Delta \omega ,s)$; however, rather than compare $p_{i}(\Delta \omega ,s)$ to actual content $p_{\text{obs},i}(\Delta \omega ,s)$ at frequency Δω (which may not be present with sufficient signal-to-noise ratio in the recorded signal), we instead compare $p_{i}(\Delta \omega ,s)$ to ${\bar{p}}_{\text{obs},i}(\Delta \omega )$ resulting from Eq. (2). The process used to generate ${\bar{p}}_{\text{obs},i}(\Delta \omega )$ from existing data is very similar to what is used in frequency-difference beamforming (Abadi *et al.*, 2012) to overcome the same lack of low frequency content in existing signals.

## 3. Methods

For all the datasets described in Secs. 3.1 and 3.2, we reconstructed three different sound speed images.

The first reconstruction is performed by applying FWI with a homogeneous starting model (usually at a constant 1480 m/s, but there is one exception in Sec. 3.2.2). As with our prior work (Ali *et al.*, 2024), this application of FWI reconstructs the sound speed and the attenuation in two passes, each of which is done from low-frequency to high-frequency. On the first pass, FWI only updates the sound speed model at each frequency. On the second pass, FWI alternates between updating the sound speed and the attenuation at each frequency. Each update to either the sound speed or the attenuation is done with 3 iterations of the conjugate gradient method. For each dataset, the lowest frequency used in the FWI reconstruction is not low enough to overcome cycle skipping from the homogeneous starting model. In Secs. 3.1 and 3.2, we specify the schedule of frequencies used in the two-pass FWI for each dataset.

The second reconstruction is performed using the frequency differencing technique to extrapolate low frequency content below the lowest frequency used in the first application of FWI. In Secs. 3.1 and 3.2, we specify which frequencies were generated by frequency differencing. FWI is then used to reconstruct the sound speed image based on the data generated by frequency differencing. However, this FWI is not used to reconstruct the attenuation map and is done in only a single pass from the lowest to the highest frequency generated by frequency differencing. Another key difference between this technique and the first reconstruction is that this FWI only applies two iterations of the conjugate gradient to update the sound speed at each frequency. The purpose of this reconstruction is to replace the homogeneous starting model used in the first reconstruction so that our previous two-pass FWI can overcome cycle skipping.

The third and final reconstruction is performed using the result of the second reconstruction as an initial model for the two-pass FWI algorithm used in the first reconstruction. By using the result of frequency differencing in lieu of the homogeneous starting model, our previous two-pass FWI will be shown to overcome cycle skipping without changing which frequencies are used in the FWI.

### 3.1 Numerical simulations

The open-source k-Wave (Treeby and Cox, 2010) toolbox was used to simulate single-element transmissions (1.0 MHz center frequency; 75% fractional bandwidth) from a ring array (22 cm diameter; 512 elements). The numerical phantom used for the sound speed and attenuation maps in the simulation was based on the matlab built-in modified Shepp-Logan phantom. Received channel data (time traces for the received signal from each individual element for each single-element transmission) from these k-Wave simulations were used to reconstruct the sound speed and attenuation in the medium using FWI. No noise was added to the simulated channel data.

For our simulated data, the two-pass FWI used frequencies ranging from 0.6 to 1.25 MHz in 50 kHz steps on the first pass, and 0.625 to 1.275 MHz in 50 kHz steps on the second pass. The first reconstruction used a homogeneous 1480 m/s sound speed as the starting model. To improve our starting model, frequency differencing was used to extrapolate data from 0.15 to 0.95 MHz (in 50 kHz steps) solely based on the simulated data from 0.6 to 5.0 MHz. We report the root mean square error (RMSE), peak signal-to-noise ratio (PSNR), and structural similarity index measure (SSIM) (Hore and Ziou, 2010) of each sound speed reconstruction.

### 3.2 Experiments

For each experiment described in the following, a 22 cm diameter ring transducer with 1024 elements (Transducer Works, Centre Hall, PA) attached to a Verasonics Vantage 256 scanner via a UTA1024-MUX connector (Verasonics Inc., Kirkland, WA) was used to collect receive channel data from all 1024 elements for each single-element transmit (2.5 MHz center frequency) on the ring, resulting in a full 1024 × 1024 matrix capture of time series channel data (sampled at 12.5 MHz). Using the UTA1024-MUX, the acquisition sequence consisted of single-element transmissions, repeated four times for each 256-element subaperture of receivers (elements 1–256, 257–512, 513–768, and 769–1024), resulting in a total of 4096 transmit events to collect the full 1024 × 1024 matrix capture.

#### 3.2.1 In vitro breast phantom

The same Yezitronix multi-modality breast phantom model B-MM-1.2 (Yezitronix Group Inc., Saint-Laurent, QC, Canada) used in our prior work (Ali *et al.*, 2024) was used to demonstrate the frequency differencing method in this work. Two different slices of the phantom were imaged. In these particular acquisitions, we lacked sufficient signal below 0.75 MHz for FWI due to the low gain setting during the acquisition. Therefore, the usable data from this experiment is restricted to the range of 0.75 to 4.0 MHz. However, in the experiments described later in Secs. 3.2.2 and 3.2.3, this gain setting was refined to produce higher signal levels below 0.75 MHz.

For the data coming from this *in vitro* breast phantom, our two-pass FWI used frequencies ranging from 0.75 to 1.0 MHz in 50 kHz steps on the first pass, and 0.775 to 1.025 MHz in 50 kHz steps on the second pass. The first reconstruction used a homogeneous 1480 m/s sound speed as the starting model, and to improve our starting model, frequency differencing was used to generate data from 0.1 to 0.55 MHz (in 50 kHz steps) solely based on the data acquired from 0.75 to 4.0 MHz.

#### 3.2.2 Ex vivo human brain preserved in alcohol

We imaged an *ex vivo* human cadaveric brain sample, which was embalmed and subsequently preserved in a 50% ethanol/water solution for one year. Due to this preservation process, the sound speed inside the brain ranged from 1600 to 1700 m/s well above the range expected *in vivo*. The preserved brain was placed inside a plastic bag filled with de-ionized water immediately before imaging. The large contrast in sound speed between the preserved brain (1625–1700 m/s) and the water background (1480–1500 m/s) was a significant challenge for FWI. To accommodate the high sound speed of the preserved brain, a homogeneous starting model with an elevated 1600 m/s sound speed was used in FWI. In these experiments, our gain settings allowed us to acquire data down to 0.45 MHz, which further mitigates the impact of cycle skipping. The brain was oriented such that we could acquire data from both an axial and a sagittal slice of the brain.

For the data acquired from the preserved human brain, our two-pass FWI used frequencies ranging from 0.45 to 1.0 MHz in 50 kHz steps on the first pass, and 0.475 to 1.025 MHz in 50 kHz steps on the second pass. To improve upon our homogeneous 1600 m/s starting model, frequency differencing was used to generate data from 0.1 to 0.55 MHz (in 50 kHz steps) solely based on the data acquired from 1.0 to 5.0 MHz.

#### 3.2.3 Ex vivo macaque brain inside replica human skull

A macaque brain, preserved in a 10% formalin solution and rehydrated in a plastic bag filled with 10 mM PBS (pH 7.4) for two weeks prior to imaging, was placed inside a replica human skull (Skulls Unlimited Inc., Oklahoma City, OK) characterized at approximately 2450 m/s. The purpose of this experiment was to demonstrate the impact of frequency differencing in a difficult transcranial imaging scenario. Data were acquired from two different axial slices of the macaque brain.

For the data collected in this experiment, our two-pass FWI used frequencies ranging from 0.55 to 1.0 MHz in 50 kHz steps on the first pass, and 0.575 to 1.025 MHz in 50 kHz steps on the second pass. The first reconstruction used a homogeneous 1480 m/s sound speed as the starting model, and to improve our starting model, frequency differencing was used to generate data from 0.1 to 0.9 MHz (in 50 kHz steps) solely based on the data acquired from 1.0 to 5.0 MHz.

## 4. Results and discussion

### 4.1 Demonstration in numerical simulations

**Fig. 1.**
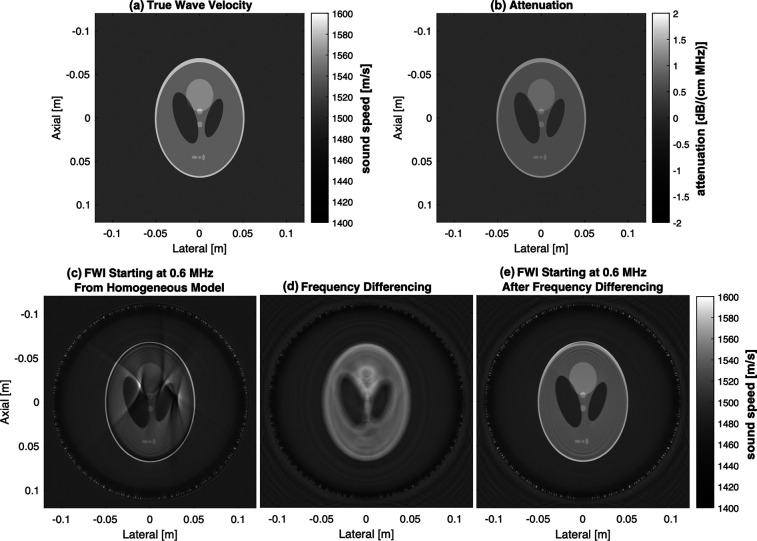
Demonstration of frequency differencing on simulated channel Data. (a) and (b) Sound speed and attenuation of the modified Shepp-Logan phantom used in the k-Wave simulation. (c) FWI applied to simulated data starting at 0.6 MHz from a homogeneous 1480 m/s sound speed model. (d) FWI applied to data synthesized by frequency-differencing. (e) FWI applied to simulated data starting at 0.6 MHz from the result of frequency differencing.

Figure 1 demonstrates the impact of frequency differencing in a numerical UST simulation. Figures 1(a)–1(b) show the profile of sound speed and attenuation used in the simulation. When starting from a homogeneous 1480 m/s starting model, our two-pass FWI reconstructs a sound speed profile with significant cycle skipping artifacts that obscure the features inside the Shepp-Logan phantom [Fig. 1(c)]. The RMSE, PSNR, and SSIM of this sound speed reconstruction are 18.1 m/s, 23.0 dB, and 0.82, respectively. Then in Fig. 1(d), we use frequency differencing to create low-frequency content that overcomes cycle skipping. This produces a low-resolution reconstruction of sound speed that better captures the bulk sound speed in major compartments of the phantom. The result of Fig. 1(d) is then used as a starting model for our two-pass FWI, which produces the result shown in Fig. 1(e). The RMSE, PSNR, and SSIM of the reconstructions shown in Figs. 1(d) and 1(e) are 14.6 m/s, 24.9 dB, and 0.82; and 6.7 m/s, 31.6 dB, and 0.96, respectively.

### 4.2 Validation in a breast phantom

**Fig. 2.**
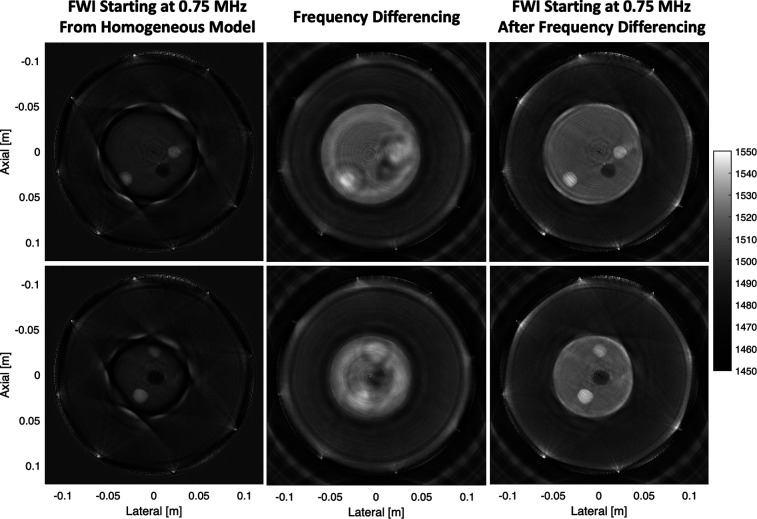
Demonstration of frequency differencing in breast phantom data. Each row of images represents a different slice of the phantom. The left column shows FWI applied to the received channel data starting at 0.75 MHz from a homogeneous 1480 m/s sound speed model. The middle column shows FWI applied to data synthesized by frequency-differencing. The right column shows FWI applied to the received channel data starting at 0.75 MHz from the result of frequency differencing.

So far, the impact of frequency differencing as a starting model for FWI has been demonstrated *in silico*. The results of Fig. 2 serve to further validate this approach with channel data acquired from a physical breast phantom. In Table 1, we report the reconstructed and expected sound speed in the background material of the phantom as well as the high sound speed lesions and fluid-filled cysts embedded inside the phantom. The expected sound speed for each material is based on values previously reconstructed for this phantom (Ali *et al.*, 2024). For each slice of the phantom, FWI is unable to reconstruct a quantitatively accurate sound speed profile when starting from the homogeneous sound speed model. However, frequency-differencing provides a much better starting model that allows FWI to accurately reconstruct the sound speed profile without cycle skipping artifacts. In each case, FWI reconstructs sound speed values closer to the previously reported values when frequency differencing is used to create the starting model.

**Table 1.** Reconstructed sound speed in breast phantom slices shown in Fig. 2.

Phantom material	Expected	FWI from homogenous model	Frequency difference (FD)	FWI after FD
Background	1510.3 m/s	1484.6 m/s	1516.7 m/s	1509.8 m/s
Lesion	1532.3 m/s	1511.4 m/s	1528.9 m/s	1530.1 m/s
Cysts	1491.6 m/s	1464.4 m/s	1498.9 m/s	1491.5 m/s

In this work, we used a 2.5 MHz center-frequency pulse, which gave us usable frequency content down to 0.75 MHz, while the prior work (Ali *et al.*, 2024) used a 0.7 MHz center-frequency pulse from the same ring array to bring out low frequency content at 0.35 MHz. Although this low-frequency pulse circumvented cycle skipping in the prior work, the excitation was well outside the normal bandwidth of the transducer, eliciting a frequency response that varied over the ring elements, rendering content at higher frequencies unusable. Ultimately, frequency differencing allows FWI to reconstruct accurate sound speed images when transmitting at the natural center frequency of the transducer.

### 4.3 Application to a preserved human brain

**Fig. 3.**
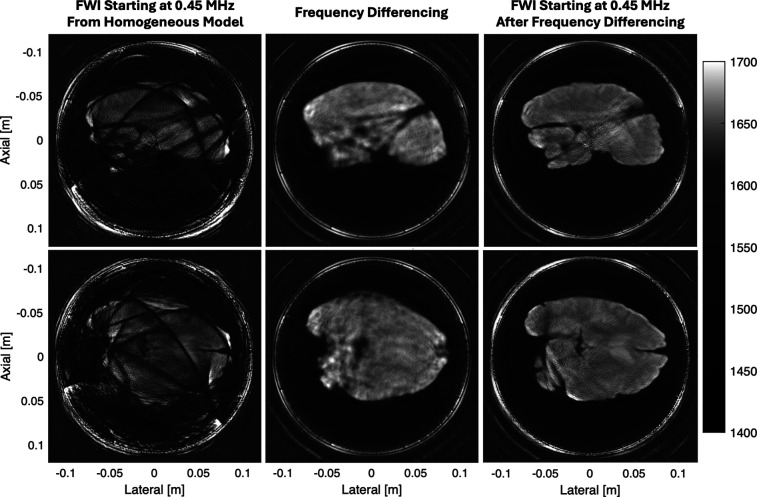
Application of frequency differencing to the preserved human brain. The top row corresponds to a sagittal slice while the bottom row corresponds to an axial slice of the brain. The left column shows FWI applied to the received channel data starting at 0.45 MHz from a homogeneous 1600 m/s sound speed model. The middle column shows FWI applied to data extrapolated by frequency differencing. The right column shows FWI applied to the received channel data starting at 0.45 MHz from the result of frequency differencing.

After demonstrating the impact of frequency differencing both *in silico* and *in vitro*, Fig. 3 shows a pair of imaging examples where frequency differencing enables the visualization of brain anatomy. The two-pass FWI starting from the homogeneous model results in major cycle skipping artifacts that obscure both the shape and the features of the brain. For both the axial and sagittal views, the frequency differencing starting model captures the bulk sound speed and shape of the brain but does not capture any detailed features. When the result of frequency differencing is used as the starting model, the two-pass FWI produces high-resolution sound speed images of the brain without any major cycle skipping artifacts. Despite the tissue preservation process that significantly changed the properties of the brain tissue, we can produce detailed images of the underlying brain anatomy.

### 4.4 Application to transcranial imaging

**Fig. 4.**
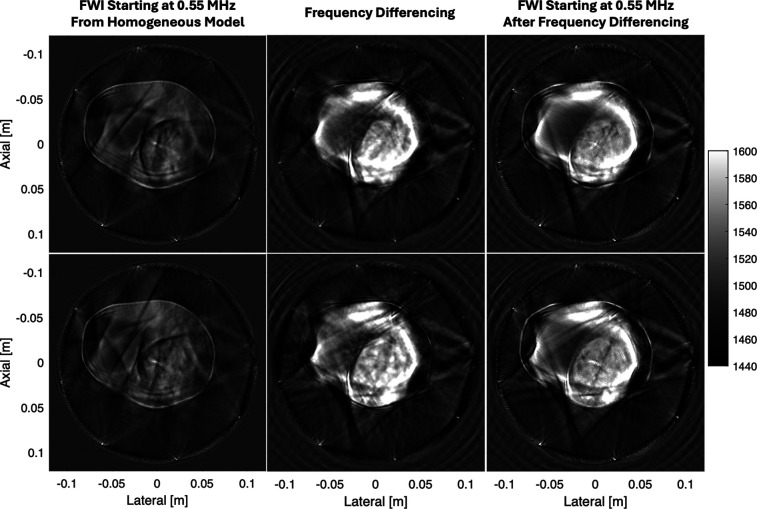
Transcranial imaging scenario. Each row of images represents a different slice of the macaque brain inside the replica human skull. The left column shows FWI applied to the received channel data starting at 0.55 MHz from a homogeneous 1480 m/s sound speed model. The middle column shows FWI applied to data synthesized by frequency-differencing. The right column shows FWI applied to the received channel data starting at 0.55 MHz from the result of frequency differencing.

Last, in Fig. 4 we demonstrate our frequency differencing approach in a challenging transcranial imaging scenario. When starting from a homogeneous model, FWI is unable to reconstruct any discernible object inside the replica skull, and the image is dominated by cycle skipping artifacts. When frequency differencing is applied, we begin to discern the shape and size of the smaller macaque brain inside the replica human skull. However, just inside the skull, frequency differencing results in a halo of high sound speed most likely caused by the strong refraction and diffraction around the skull.

As stated in Sec. 2.2, frequency differencing has an implicit linear phase assumption. For the small tissue heterogeneities in soft tissue, the deviations from this assumption are well-contained. However, when ultrasound traverses the skull, strong refractions and diffractions around the skull cause a very complex waveform to emerge on the opposite side. Frequency differencing may struggle to adequately extrapolate the low-frequency signal under these conditions, resulting in the displacement of the high sound speed in the skull to the halo artifacts seen in the images.

Despite this skull-induced artifact, frequency differencing continues to improve FWI when used as a starting model. From the resulting image, we can discern the brain and its major anatomical features (e.g., the midline, fissures, and sulci). However, the skull-induced frequency differencing artifact obscures the parts of the brain closest to the skull. Therefore, there is a need to further refine frequency differencing to better accommodate such challenging imaging targets.

## 5. Conclusions and future work

This work demonstrates that frequency differencing can synthesize the low-frequency data needed to kickstart FWI without cycle skipping. By applying FWI to the low-frequency data generated by frequency differencing, we produce a low-resolution starting model for conventional FWI. Despite its lack of spatial resolution, the frequency-differencing starting model allows FWI to converge to the desired result at higher frequencies. The spatial resolution and accuracy of the frequency differencing starting model are ultimately limited by the fundamental mismatch between the simulation model $p_{i}(\Delta \omega ,s)$ and the frequency-differenced data ${\bar{p}}_{\text{obs},i}(\Delta \omega )$. There are two possible routes to overcoming this mismatch. The first option would be to incorporate frequency differencing into the simulation model $p_{i}(\Delta \omega ,s)$ so that it accurately models the frequency difference calculation and all the frequencies used in the calculation. In other words, frequency differencing would be applied not only to the measured data but also to the simulated data. The second option would be to retain the same model used in the standard frequency-domain FWI but to modify the frequency difference calculation (potentially via deep learning) so that it better captures the physics at a single low-frequency. Both approaches would reduce the mismatch between the model and the data from opposite ends, and improve the efficacy of frequency differencing.

## Supplementary material

See the supplementary material for (1) additional comparisons to ray tomography as a starting model for FWI, and (2) detailed line profiles through each image. The computational cost of applying FWI to the low-frequency data extrapolated by frequency differencing is much greater than ray tomography. However, ray tomography is inherently less robust because of inaccuracies in time-of-flight picking. In general, the frequency-differencing starting model avoids the artifacts seen in ray tomography and better avoids cycle skipping.

## Data Availability

In order to make our efforts more accessible to the broader research community, we have provided sample code and channel data at https://github.com/rehmanali1994/FrequencyDifferencing (doi:10.5281/zenodo.13785188).
